# Use of pus metagenomic next-generation sequencing for efficient identification of pathogens in patients with sepsis

**DOI:** 10.1007/s12223-024-01134-7

**Published:** 2024-02-11

**Authors:** Zhendong Chen, Tingting Ye, Yuxi He, Aijun Pan, Qing Mei

**Affiliations:** 1 Department of Intensive Care Unit, The First Affiliated Hospital of USTC, Division of Life Sciences and Medicine, University of Science and Technology of China, Hefei, Anhui 230001 China; 2 Department of Cardiovascular Medicine, The First Affiliated Hospital of USTC, Division of Life Sciences and Medicine, University of Science and Technology of China, Hefei, Anhui 230001 China; 3 Department of Intensive Care Unit, The Affiliated Provincial Hospital of Anhui Medical University, Anhui, 230001 China; 4 WanNan Medical College, Wuhu, 241002 Anhui China

**Keywords:** Metagenomic next-generation sequencing (mNGS), Sepsis, Pus, ﻿Mixed infection, Obligate anaerobic infection

## Abstract

The positive detection rate of blood metagenomic next-generation sequencing (mNGS) was still too low to meet clinical needs, while pus from the site of primary infection may be advantageous for identification of pathogens. To assess the value of mNGS using pus in patients with sepsis, thirty-five samples were collected. Pathogen identification and mixed infection diagnosis obtained by use of mNGS or cultivation methods were compared. Fifty-three aerobic or facultative anaerobes, 59 obligate anaerobes and 7 fungi were identified by the two methods. mNGS increased the accuracy rate of diagnosing aerobic or facultative anaerobic infections from 44.4% to 94.4%; mNGS also increased the sensitivity of diagnosing obligate anaerobic infections from 52.9% to 100.0%; however, mNGS did not show any advantage in terms of fungal infections. Culture and mNGS identified 1 and 24 patients with mixed infection, respectively. For obligate anaerobes, source of microorganisms was analyzed. The odontogenic bacteria all caused empyema (n = 7) or skin and soft tissue infections (n = 5), whereas the gut-derived microbes all caused intra-abdominal infections (n = 7). We also compared the clinical characteristics of non-obligate anaerobic and obligate anaerobic infection groups. The SOFA score [9.0 (7.5, 14.3) vs. 5.0 (3.0, 8.0), P = 0.005], procalcitonin value [4.7 (1.8, 39.9) vs. 2.50 (0.7, 8.0), P = 0.035], the proportion of septic shock (66.7% vs. 35.3%, P = 0.044) and acute liver injury (66.7% vs. 23.5%, P = 0.018) in the non-obligate anaerobic infection group were significantly higher than those in the obligate anaerobic infection group. In patients with sepsis caused by purulent infection, mNGS using pus from the primary lesion may yield more valuable microbiological information.

**Supplementary Information:**

The online version contains supplementary material available at 10.1007/s12223-024-01134-7.

## Introduction

Sepsis is a systemic inflammatory response syndrome caused by pathogenic microorganisms infecting the body, often involving local purulent lesions (Li et al. 2021). Although the drainage of pus is important in treatment, identification of the etiology and the selection of appropriate antibiotics are also key to treatment (Rasane et al. 2020). Pus is a fluid accumulation formed by necrosis and liquefaction of diseased tissue after infection in body tissues, organs, or body cavities. It occurs at the site of infection in almost any organ or tissue of a patient, and specimens from this primary lesion site are considered ideal for the detection of pathogens (Wang et al. 2018). However, traditional microbiological detection methods have significant limitations regarding the etiological diagnosis of pus. For example, in the process of collecting, storing, and transporting pus specimens in many hospitals, the growth conditions for anaerobic bacteria are often ignored, resulting in a low positive detection rate using conventional detection methods (Shenoy et al. 2017). In addition, multiple pathogenic microorganisms may be present in pus samples, but real mixed infections are difficult to diagnose because of competing growth or human factors (Siqueira et al. 2013).

In recent years, metagenomic next-generation sequencing (mNGS) technology has attracted increasing attention (Simner et al. 2018). This methodology can amplify and sequence all DNA fragments within a test sample without the use of any primers or probes. In theory, mNGS can identify all potential pathogens in a single experiment, and comprehensively detect the pathogens that may be present in a specimen, thereby facilitating the early diagnosis of infection. The application of this new technology to multiple types of samples (e.g., blood, cerebrospinal fluid, bronchoalveolar lavage fluid) has been validated and approved (Geng et al. 2021; Kanaujia et al. 2022; Fang et al. 2020; Yang et al. 2022). For sepsis, previous reports have mainly focused on mNGS of blood samples (Sun et al. 2022; Yan et al. 2021; Cao et al. 2022). Although blood-mNGS offered great advantages over traditional culture methods, its positive detection rate was still too low to meet clinical needs. Analyzing specimens from the site of primary infection may be advantageous for identification of pathogens (DiEuliis et al. 2016). This study aims to use mNGS technology to more accurately understand the microbial etiology in pus, and then provide a reference for the treatment of sepsis caused by purulent infection.

## Materials and methods

### Study design and patient selection

The clinical data of sepsis patients admitted to the 130-bed comprehensive intensive care unit (ICU) of the First Affiliated Hospital of University of Science and Technology of China (USTC) from March 2019 to October 2022 were extracted from the electronic medical record system. Patients from whom pus samples were obtained and submitted for mNGS were included. Cases with specimens submitted for inspection 48 h or more after admission to the ICU, pus cultures not submitted simultaneously, incomplete medical records, and samples that fail quality control of mNGS were excluded. Demographic data, immune status, disease severity, laboratory test results, complications, treatment measures, and patient outcomes were collected for each enrolled patient. According to the etiological diagnosis results, the patients were divided into the obligate anaerobic infection group and the non-obligate anaerobic infection group.

### Specimen collection

Different collection methods were used depending on the anatomical characteristics of the infection site. For example, bile pus, thoracic pus, and abdominal pus were generally collected through a drainage tube or puncture inserted during surgery, whereas deep soft tissue pus was collected by incision or puncture. All samples were divided into two after collection, with one of these specimens being delivered to a laboratory for further analysis within 1 h. Traditional etiological identification methods include aerobic bacterial culture, anaerobic bacterial culture, and fungal culturing procedures. Each sample was split into both aerobic and anaerobic vials containing modified tryptic soy broth (TSB) (1:10 dilution). Aerobic cultures (5% CO2) were incubated with 200 rpm shaking, while anaerobic cultures (80% N2, 10% H2 and 10% CO2) were incubated statically. Culturing was performed at 37 °C for 72 h both aerobically and anaerobically. Czapek’s agar and Sabouraud’s agar (Beijing AoBoXing Bio-Tech Co., Ltd., China) were used for fungal culture. It was incubated at 30 °C for 96 h or until a mycelial mat formed. Successfully cultured strains were identified using the Vitek MS system (BioMerieux).

### Metagenomic next-generation sequencing analysis

The other specimen, described above, was sent to Jieyi Biotechnology Co., Ltd. for quantitative mNGS. After receiving the sample, 1.20 mL of the sample was added to a shaking tube in a homogenizer. Following homogenization, the sample was centrifuged at 12,000 rpm for 3 min. Then, working in a biological safety cabinet, 400 μL of the homogenized sample was placed into the cassette of the NGSmaster™ automated workstation according to the standard operating procedures. The NGSmaster™ automated workstation performs automated nucleic acid extraction, nucleic acid fragmentation, end repair, end adenylation (of the A tail at the 3ʹ end), and the addition of sequencing adapters. After ligation and purification, a sequencing library was formed. The library was quantified by real-time PCR, then shotgun sequenced using the Illumina Nextseq™ high-throughput sequencing platform.

After filtering out low-quality and low-complexity data using fastp (https://github.com/OpenGene/fastp), approximately 20 million 75 bp single-end reads were generated for each library. Informatics analysis was conducted using bowtie2 software (http://bowtie-bio.sourceforge.net/bowtie2/index.shtml) to filter the human genome sequence data (GRCh38.p13) and Kraken software (https://github.com/DerrickWood/kraken) to align the remaining sequence data with reference sequences from bacteria, fungi, viruses, and parasites in microbial reference databases (*NCBIGenBank* and in-house curated microbial genome data) to determine the microbial species and relative abundances. The alignment results were further verified using the NCBI BLAST software (https://blast.ncbi.nlm.nih.gov/Blast.cgi). Two negative controls (~ 10^4^ and 10^6^ human immortalized cells) and a positive control (containing a mixture of killed bacteria, fungi, and pseudovirions) were included.

### Gold standard for causative pathogens

The final determination of the causative pathogen (the gold standard) was based on comprehensive clinical diagnostic criteria, which have been determined by comprehensive analysis of microbiological results and other relevant information (such as clinical features, laboratory tests, imaging studies, and the observation of treatment effects). Microorganisms detected by culture or mNGS but not considered causative pathogens according to the gold standard were defined as false positives. Two infection management experts (QM and AP) independently reviewed each patient's electronic medical records and resolved any disagreements through in-depth discussions.

### Definitions

The impact of mNGS results on clinical adjustment of antimicrobial therapy was divided into four elements, among which initial targeted therapy and de-escalation therapy were considered clinically beneficial drug adjustments (Zhan et al. 2021). The other two elements were determining that the original treatment was reasonable and that it was not helpful for pathogen diagnosis or drug treatment. These determinations were made independently by two intensivists through a comprehensive assessment of a patient’s clinical manifestations, laboratory tests, and microbiological examinations (culture and mNGS). If there remained disagreement between the two intensivists after in-depth discussions and no consensus could be reached, another senior intensivist was consulted.

### Statistical analysis

Data conformance to normal distribution is described by mean ± standard deviation (SD), the independent sample *t* test was used to compare the measurement data between groups. Continuous variables that do not conform to a normal distribution were represented by the median and quartile, and the Mann–Whitney U rank-sum test was used to compare two independent samples. The enumeration data were expressed by the ratio or rate, and the comparison of two independent samples was performed by the chi-square test or Fisher's exact test. We used the results of the gold standard as a reference standard to calculate the diagnostic efficacy of culture and mNGS methods, and established a fourfold table to calculate sensitivity, specificity, the positive predictive value, and the negative predictive value using McNemar’s paired chi-square test. Statistical analysis was performed using IBM SPSS version 26.0 software, all results were two-tailed, and differences were considered statistically significant with a P value < 0.05.

## Results

### Patient characteristics

During the study period, 246 patients with sepsis were admitted to the ICU, 42 of whom had pus samples collected and sent for culture and mNGS. Seven patients were excluded because the interval between sample culture and mNGS was more than 24 h, and 35 patients were finally included in the study. Table 1 presents the clinical characteristics of these patients. Sum of all comorbidities is higher than 100%, which is caused by more comorbidities in some of the patients. For example, hypertension, digestive system disease and diabetes are the three most common complications in this study, which were often combined in the same patient. Sepsis caused by intra-abdominal infection (37.1%) was the most common among patients included in this study, followed by thoracic (28.6%) and skin and soft tissue (22.9%) infections. Upon ICU admission, the acute physiology and chronic health evaluation II (APACHE II) and sequential organ failure assessment (SOFA) scores were 19.1 ± 4.6 and 8 (4, 10), respectively. Thirty-four (97.1%) patients had already received anti-infective therapy at the time of mNGS. Nine (25.7%) patients died in the ICU.

**Table 1:** Clinical characteristics of the 35 patients included in this study

Characteristics	35 patients
Age, years, mean ± SD	55.5 ± 14.8
Gender, female, n (%)	10 (28.6)
**Comorbidities, n (%)**	
Immunosuppression	3 (8.6)
Diabetes	8 (22.9)
Tumor	6 (17.1)
Hypertension	12 (34.3)
Cardiac insufficiency	7 (20.0)
Digestive system diseases	10 (28.6)
Surgical history	13 (37.1)
Other^a^	2 (5.7)
**The site of infection, n (%)**	
Abdominal cavity	13 (37.1)
Thoracic cavity	10 (28.6)
Skin soft tissue	8 (22.9)
Other^b^	4 (11.4)
**Severity of illness at ICU admission**	
APACHE II score, mean ± SD	19.1 ± 4.6
SOFA score, median (IQR)	8 (4, 10)
**Treatment**	
Mechanical ventilation, n (%)	23 (65.7)
Vasoactive drug use, n (%)	18 (51.4)
**Outcomes**	
Length of ICU stay, days, median (IQR)	7.4 ± 5.5 (3, 11)
ICU death, n (%)	9 (25.7)
Death within 28-days, n (%)	10 (28.6)

^a^Others, including one case each of rheumatic immune system disease and stroke

^b^Others, including gallbladder (n = 3) and joint cavity (n = 1)

### Identification of Pathogens using the mNGS and culture methods

For culture methods, a positive result was reported in 19 patients (54.2%), whereas the mNGS results were positive in all 35 patients. A total of 125 potential causative pathogens were reported in 35 pus samples by mNGS, of which three were considered false positives, including two cases of *Ralstonia* and one case of *Mycobacterium avium*, which were considered contamination. Although mNGS reported the presence of one case of Epstein-Barr virus and one case of Cytomegalovirus in pus specimens from two patients, neither was considered a causative pathogen. Culture detection methods revealed 29 pathogens among 20 patient samples, of which six were considered false positives, including one *Klebsiella pneumoniae*, one *Streptococcus anginis*, and one *Aeromonas hydrophila*, which were assumed to be species identification errors, and one strain of *Escherichia coli*, one strain of *Acinetobacter baumannii*, and one strain of *Sphingomonas paucimobilis*, which were considered to be contamination. After comprehensive analysis, a total of 120 pathogenic bacteria were identified by the final gold standard, including 112 bacterial strains (53 aerobic or facultative anaerobes, 59 obligate anaerobes) and seven fungi. In addition, one strain of oral *Mycoplasma* was detected.

**Fig. 1:**
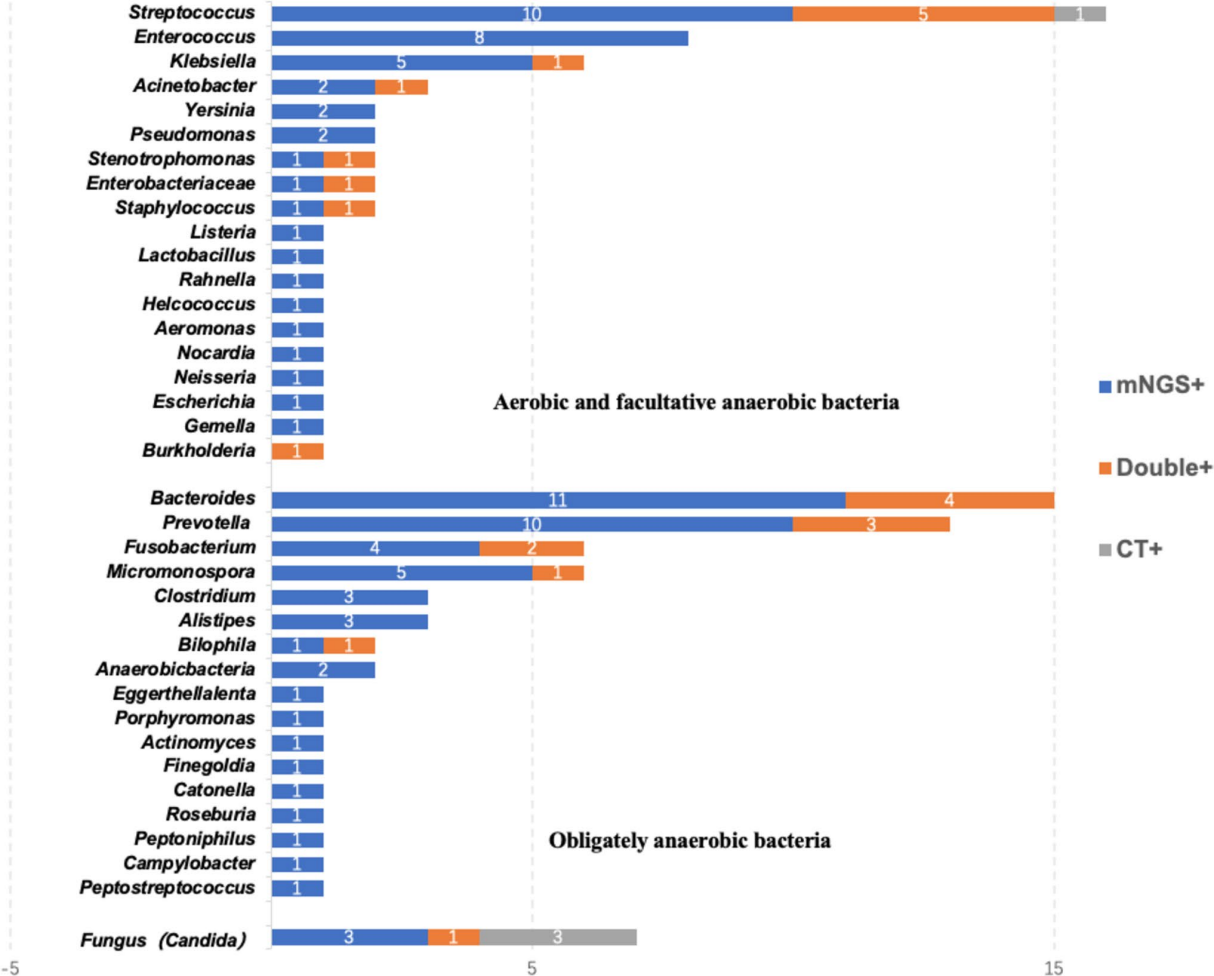
﻿The overlap of positivity between mNGS technique and culture method for different pathogens. Due to the excessive classification at the species level, the genus level is used for classification. Blue indicates detection by mNGS only, gray indicates detection by culture method only, and orange indicates detection by both methods simultaneously. *The pathogens were observed to have a higher positive rate by mNGS than that by culture method, and the difference was significant (P < 0.05)

Figure 1 lists all bacteria and fungi (at the genus level) identified by the gold standard. Bacteria were detected by mNGS in all specimens except for one *Streptococcus*, whereas culture detected only 23 out of 112 bacterial strains. There was a statistically significant difference in the positive rate of bacterial detection between the two methods (P < 0.001). Among aerobes and facultative anaerobes, *Streptococcus* was the most frequently detected bacterial genus (n = 16, 30.2%), followed by *Enterococcus* (n = 8, 15.1%) and *Klebsiella* (n = 6, 11.3%). Specifically, the positive rate of mNGS in detecting *Streptococcus* (93.8% vs 37.5%; P = 0.012) and *Enterococcus* (100.0% vs 0.0%; P = 0.021) was higher compared with culture methods. Although the difference was not statistically significant, mNGS detected all *Klebsiella*, whereas the culture method only detected 1 of 6 patients. Only 11 obligate anaerobes were detected by the culture method, whereas all of the obligate anaerobes were reported by mNGS. Among them, *Bacteroides* was the most frequently detected bacterial genus (n = 15, 25.4%), followed by *Prevotella* (n = 13, 22.0%). Specifically, the positive rate of mNGS in detecting *Bacteroides* (100.0% vs. 26.7%; P = 0.026), *Prevotella* (100.0% vs. 23.1%; P = 0.044) and *Fusobacterium* (100.0% vs. 33.3%; P = 0.025) was higher compared with culture methods. The seven fungi detected were all *Candida* species (six *Candida albicans* and one *Candida glabrata*). mNGS detected three strains of *Candida*, culture also detected three strains of *Candida*, but only one case of *Candida* was reported by the two methods at the same time, and the rates of detection did not show a statistically significant difference (P = 1.000).

### Diagnostic performance comparison of mNGS and culture

**Fig. 2:**
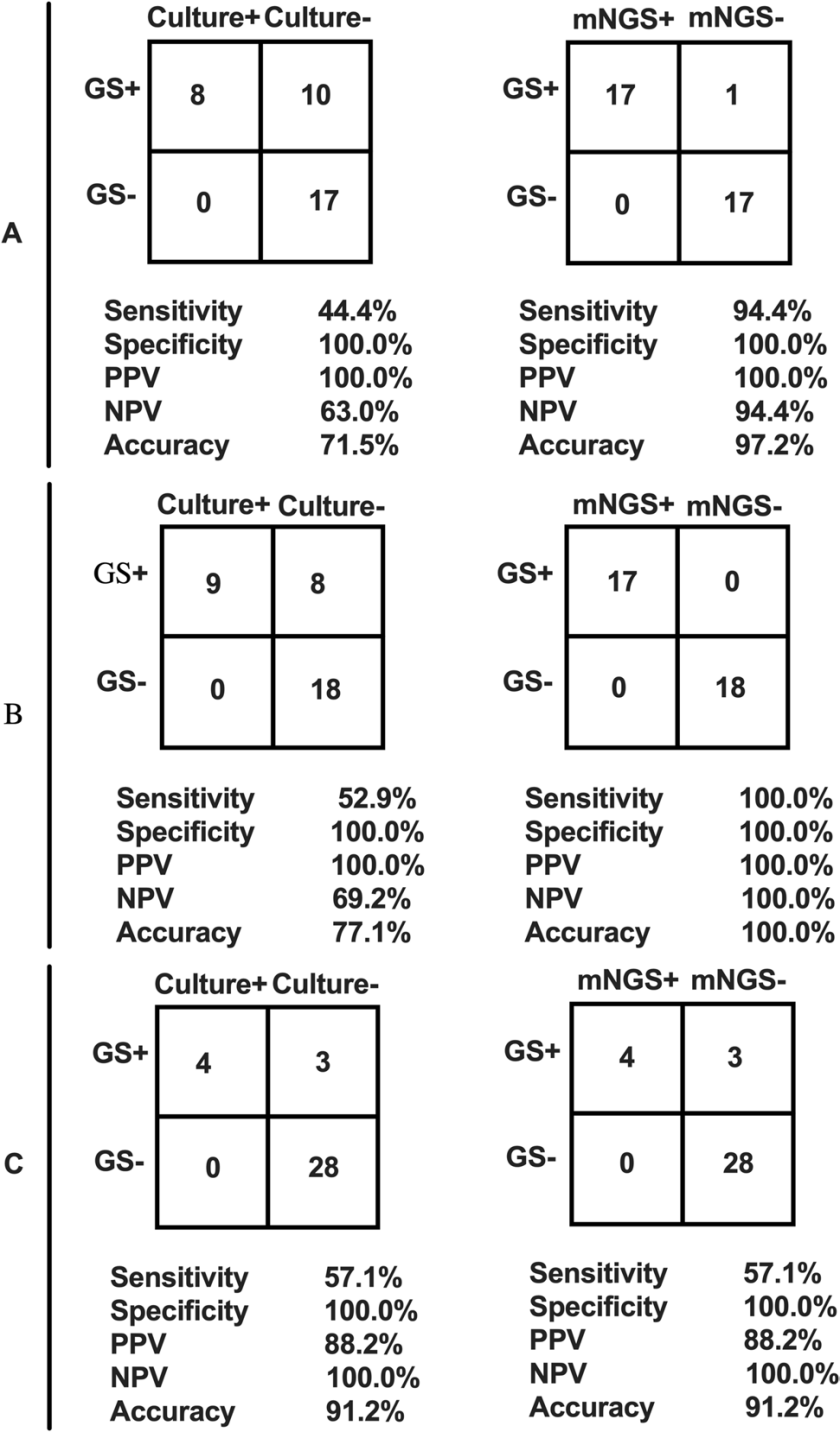
Diagnostic performance comparison of mNGS and culture. Two by 2 contingency tables showing the diagnostic performance of mNGS and culture method with gold standard results as the reference standard for aerobic and facultative anaerobic infection (**A**), obligate anaerobic infection (**B**) and fungal infection (**C**). Accuracy refers to how many ratios are correct in all judgments, calculated using the formula (True Postive + Ture Negative True Positive + Ture Negative + False Positive + False Negative). GS, gold standard; PPV, positive predictive value; NPV, negative predictive value

Using the gold standard results as a reference, the diagnostic efficacies of mNGS and culture methods for aerobic or facultative anaerobic, obligate anaerobic, and fungal infections were compared (Fig. 2). It was found that mNGS increased the sensitivity of diagnosing aerobic or facultative anaerobic infections from 44.4% to 94.4%, and the accuracy rate increased from 71.5% to 97.2%; mNGS also increased the sensitivity of diagnosing obligate anaerobic infections from 52.9% to 100.0%, and the accuracy rate increased from 77.1% to 100.0%. Moreover, the specificity of mNGS remains at 100%, irrespective of whether they are bacteria or fungi. However, mNGS did not show any advantage in terms of fungal infections.

### Mixed infection identified by mNGS and culture

Infection with more than one pathogen is defined as a mixed infection. Out of the 35 patients, culture diagnosed a mixed infection in only one patient, whereas mNGS identified two (5.7%) patients with two infecting pathogens, seven (20.0%) patients with three infecting pathogens, and 15 (42.9%) patients with at least four infecting pathogens (Supplementary Table 1). Patients can be infected with up to nine pathogens. The most common type of mixed infection is bacteria-bacteria, followed by bacteria-fungus. In addition, 15 patients (42.9%) had a mixed infection comprising non-obligate anaerobes and obligate anaerobes.

### Concordance between the mNGS and microbiological culture methodologies

**Fig. 3:**
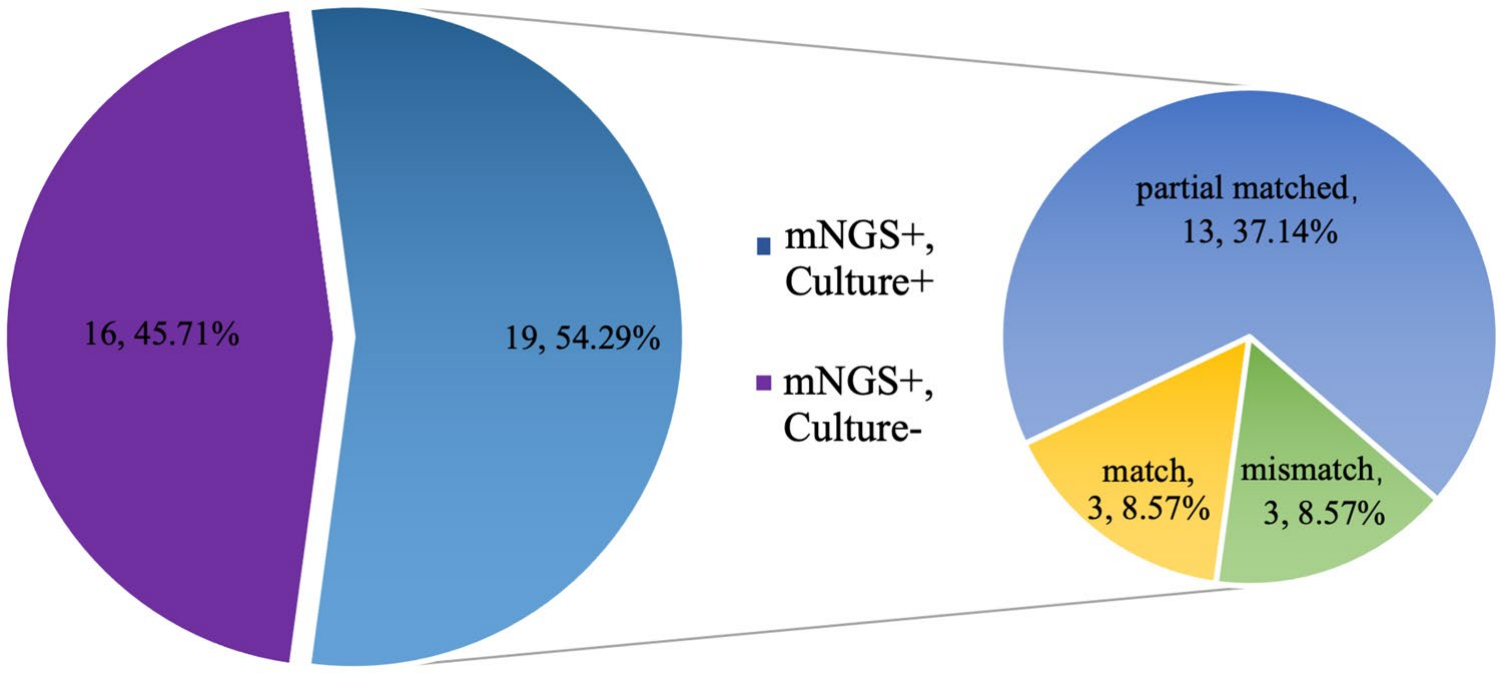
Concordance analysis between mNGS and culture method for bacterial and fungal detection. For the double-positive subset, the results of the two methods were divided into completely matched, partial matched (at least one pathogen detected by the two methods overlapped), and completely mismatched

For bacterial and fungal detection, mNGS and culture methods both gave positive results in 19/35 (54.3%) cases. For three patients, consistent results were obtained with both detection methods, whereas completely inconsistent results were obtained for 3 patients. For the remaining 13 patients, “partially matched” results were obtained, indicating that at least one pathogen was detected by both methods (Fig. 3).

### Impact on antibiotic treatment

Twelve patients underwent antibiotic adjustment after the mNGS results were obtained, all of which were beneficial adjustments, with nine patients initiating targeted therapy and the remaining three patients successfully de-escalating therapy (Supplementary Table 2).

### Source of microorganisms

Some bacteria (especially obligate anaerobes) have specific natural habitats in the human body. We observed that site-specific bacteria tended to be detected in pus in groups and inferred from this that the source of the infectious bacteria could be identified in 20 patients, including 12 cases of odontogenic infection, seven cases of gut-derived infection, and one case was infected by bacteria from vagina. Interestingly, the odontogenic bacteria all caused empyema (n = 7) or skin and soft tissue infections (n = 5), whereas the gut-derived microbes all caused intra-abdominal infections.

### Comparison of sepsis caused by obligate and non-obligate anaerobic bacteria

Among the 35 patients, 17 patients were mainly infected with obligate anaerobic bacteria. Using patients infected with non-obligate anaerobes (n = 18) as controls, we compared the disease severity at ICU admission, treatment course, and prognosis between the two groups (Table 2). The results showed that there was no significant difference in white blood cell count, neutrophil percentage, and C-reactive protein between the two groups at admission, but the SOFA score [9.0 (7.5, 14.3) vs. 5.0 (3.0, 8.0), P = 0.005] and procalcitonin value [4.7 (1.8, 39.9) vs. 2.5 (0.7, 8.0), P = 0.035] in the non-obligate anaerobic infection group were significantly higher than those in the obligate anaerobic infection group. The proportion of septic shock (66.7% vs. 35.3%, P = 0.044) and acute liver injury (66.7% vs. 23.5%, P = 0.018) in the non-obligate anaerobic infection group was higher than that in the obligate anaerobic infection group. In addition, the ICU mortality (38.9% vs. 11.8%, P = 0.121) and 28-day mortality (44.4% vs. 11.8%, P = 0.06) of patients in the non-anaerobic group were also higher than those in the anaerobic group, but the difference was not statistically significant.

**Table 2:** Comparison of clinical characteristics of non-obligate anaerobic infection and obligate anaerobic infection groups

Characteristics	Non-obligate anaerobic infection group (n = 18)	Obligate anaerobic infection group (n = 17)	P value
Age, years, mean ± SD	55.1 ± 13.4	55.8 ± 16.5	0.889
Gender, female, n (%)	5 (27.8)	5 (29.4)	1.000
**Severity of illness at ICU admission**			
APACHE II score, mean ± SD	19.7 ± 5.1	18.5 ± 4.1	0.501
SOFA score, median (IQR)	9.0 (7.5, 14.3)	5.0 (3.0, 8.0)	0.005
**Infection index at ICU admission**			
White blood cell count, ×10^9^/L, median (IQR)	9.6 (5.8, 18.9)	10.9 (9.0, 14.6)	0.458
C-reactive protein, mg/L, median (IQR)	101.4 (58.0, 243.9)	161.95 (106.9, 231.8)	0.171
Procalcitonin, ng/mL, median (IQR)	4.7 (1.8, 39.9)	2.5 (0.7, 8.0)	0.035
**Complications, n (%)**			
Shock	12 (66.7)	6 (35.3)	0.044
Acute lung injury	9 (50.0)	14 (82.4)	0.075
Acute kidney injury	11 (61.11)	7 (41.2)	0.318
Acute liver injury	12 (66.67)	4 (23.5)	0.018
Coagulation disorders	8 (44.44)	4 (23.5)	0.289
Encephalopathy	6 (33.3)	3 (17.7)	0.443
Multiple organ dysfunction syndrom	10 (55.6)	10 (58.8)	1.000
**Prognosis**			
Length of ICU stay, days, median (IQR)	8.0 (3.0, 11.8)	6.0 (2.5, 8.5)	0.274
ICU death, n (%)	7 (38.9)	2 (11.8)	0.121
Death within 28-days, n (%)	8 (44.4)	2 (11.8)	0.06

## Discussion

In this study, the positive detection rate obtained when using pus for mNGS reached 100.0% (35/35), compared with 54.3% (19/35) using culture methods. In terms of the number of detected pathogens, mNGS resulted in more than five times the number of pathogens detected by culture methods. This is mainly because mNGS targets nucleic acids in samples and is highly sensitive to them. Thus, mNGS offers a higher positive rate of detection of both anaerobic and aerobic bacteria. mNGS demonstrates an elevated level of sensitivity while maintaining its inherent specificity. This may be because of the infection site that originally belonged to sterile environment in this study, so the detected pathogenic microorganisms have important clinical significance. Irrespective of whether they are anaerobic or aerobic bacteria, mNGS significantly enhances the accuracy of bacterial detection, owing to its concurrent possession of satisfactory sensitivity and specificity. It may therefore be concluded that the efficiency of mNGS in diagnosing the bacterial pathogens in pus has surpassed traditional culture methods. However, fungi may also be involved in the formation of pus. In our study, four strains of *Candida* were detected by both mNGS and culture methods; therefore, mNGS did not show any advantage regarding the detection of fungi. This has also been reported for other specimens (Geng et al. 2021; Fang et al. 2020; Yang et al. 2022). DNA extraction from thick-walled microorganisms is difficult and, for this reason, the detection of fungi by DNA-targeted mNGS is a challenge (Mitchell et al. 2019). Fortunately, with the development of double-pass strong wall-breaking technology and enrichment technology of mechanical homogenization and enzymatic hydrolysis, as well as the continuous optimization of sequencing depth, this challenge may be overcome (Han et al. 2019).

Based on the unbiased mNGS data, the culture results, and the clinical characteristics of the patients, 24 of the 35 sepsis patients were diagnosed with mixed infections, with only nine patients being diagnosed with single pathogen infections. Many patients have a mixture of aerobes, facultative anaerobes, and obligate anaerobes in their pus. Recently, Böttger et al. confirmed, using 16S rRNA sequencing technology, that abscesses are not usually caused by a single microorganism, but instead involve a group of related microbiotas (Böttger et al. 2020). Therefore, a comprehensive and accurate understanding of the composition of microbes in pus will aid timely and appropriate antibiotic therapy to target the infection foci. Among the 112 strains detected in this study, 59 strains (52.7%) were obligate anaerobic bacteria, and nearly half of the patients were mainly infected with this type of bacteria, which suggests that obligate anaerobic bacteria play an important role in purulent infections. It should be noted that many clinicians and laboratory physicians were unaware of the need for anaerobic inspection, and therefore samples were processed in the laboratory under aerobic conditions. This unfortunately meant that almost all of the obligate anaerobic bacteria would have been missed by culturing. In fact, a culture environment suitable for the growth of all microorganisms is provided clinically and even if anaerobic culturing is adopted in the laboratory, the lack of necessary nutrient factors in the medium, the mutual inhibition between microorganisms, and metabolic dependence may be reasons why some organisms are missed (Böttger et al. 2021). Therefore, traditional culture methods may have distorted our knowledge of pus-related etiology, and molecular diagnosis-based methods are more suitable for the diagnosis of pus-related etiology.

In this study, we compared the severity and prognosis of sepsis caused by obligate versus non-obligate anaerobes. Interestingly, patients in the obligate anaerobe group had significantly lower SOFA scores and PCT values on ICU admission than those in the non-anaerobic group; the incidence of shock and acute liver injury is also lower, which seemed to suggest that the severity of sepsis caused by obligate anaerobes is less serious. In fact, almost all obligate anaerobic infections in this study were caused by oral or intestinal commensal bacteria, therefore representing endogenous infections, and these commensal anaerobic bacteria were considered to be relatively low-virulence microorganisms (Marik 2003). There is also evidence that obligate anaerobic bacteria are significantly more resistant to conventional antibiotics than the aerobic or facultative anaerobic bacteria that are widespread in hospitals (Sudhaharan et al. 2018; Kang et al. 2019). Therefore, patients with an obligate anaerobic infection may have more options regarding anti-infective treatment.

The oral cavity is known to constitute a reservoir of rich and varied bacterial flora. For example, *Porphyromonas pulposus*, *Nucleobacillus nucleatum*, and *Peptostreptococcus anaerobius* are often parasitic in the oral cavity (Wade 2021). Some opportunistic pathogenic microorganisms can transfer from the oral cavity to other sites (e.g., heart, brain, and skin and soft tissues) to cause abscesses (Parahitiyawa et al. 2009). However, this process does not occur via simple diffusion, and some particular bacterial communities are thought to be important in the process. For example, *Prevotella*, *Micromonas*, and *Fusobacterium* are considered to be more likely to form a complex and spread to distant tissues (Sanghavi et al. 2014). It has been reported that individual members of the group produce metabolites that are essential for the growth of other microorganisms in the group, enabling the interdependent and synergistic metabolism of a variety of microorganisms (Singh et al. 2014); however, the specific mechanisms involved are poorly understood. We found that some odontogenic microorganisms caused empyema and skin and soft tissue infections in 12 patients by analyzing the source of the pathogenic bacteria. Although odontogenic sepsis has been reported on occasion (Mannan et al. 2021), its prevalence of 34.4% in this study is higher than expected, and these patients had chronic or acute periodontal infection. This should be of concern to dentists, as early recognition and rational intervention can effectively prevent odontogenic infections from progressing to the dangerous stage of sepsis.

## Conclusion

Although this is a retrospective study with a small sample size, it effectively highlights the great advantage of mNGS in the detection of pathogenic microorganisms in pus, especially in the detection of obligate anaerobic bacteria and the diagnosis of mixed infections, for which it shows higher sensitivity than traditional culture methods. In patients with sepsis caused by purulent infection, mNGS using pus from the primary lesion may yield more valuable microbiological information. Even if mNGS is not considered convenient, the need to develop culture technology for anaerobic bacteria cannot be ignored based on our findings.

## Supplementary Information

Below is the link to the electronic supplementary material.

Supplementary file1 (DOCX 21 KB)

## Data Availability

Data generated or analysed during this study are included in this published article [and its supplementary information files], or available from the corresponding author on reasonable request.
