# Supplementary material for: Use of pus metagenomic next-generation sequencing for efficient identification of pathogens in patients with sepsis
Source: Folia Microbiol (Praha). 2024 Feb 11;69(5):1003–11. doi: 10.1007/s12223-024-01134-7 (PMC11379781; doi:10.1007/s12223-024-01134-7)
Supplement: Supplementary file 1 — Supplementary file1 (DOCX 21 KB) [file 12223_2024_1134_MOESM1_ESM.docx]

| **Supplementary Table 1**. The pathogenic microorganisms identified by mNGS in mixed infections. | | | | | | |
| --- | --- | --- | --- | --- | --- | --- |
| **Patient ID** | **Age（years）** | **Sex** | **Site of infection** | **Bacteria** | **Fungus** | **Number of pathogens** |
| P16 | 60 | M | Thorax | *Prevotella oris + peptostreptococcus stomatis + Streptococcus mitis+ Anaeroglobus geminatus +Finegoldia magna+Fusobacterium nucleatum+Dialister pneumosintes+Mycoplasma orale+bacteroides vulgatus* |  | 9 |
| P7 | 42 | F | Abdominal | *Anaerococcus lactolyticus+Peptoniphilus senegalensis+Peptostreptococcus anaerobius+Prevotella bivia+Bilophila wadsworthia+Dialister micraerophilus* |  | 6 |
| P14 | 62 | M | Abdominal | *Bacteroides thetaiotaomicron+bacteroides fragilis+Alistipes+Fusobacterium varium+Bilophila wadsworthia+klebsiella pneumoniae* |  | 6 |
| P17 | 40 | M | Skin soft tissue | *Prevotella intermedia+Streptococcus constellatus+prevotella oris+bacteroides fragilis+Micromonas parvum+peptostreptococcus stomatis* |  | 6 |
| P15 | 62 | M | Skin soft tissue | *Prevotella intermedia+Fusobacterium nucleatum+Streptococcus oralis+Rothia mucilaginosus+Gemella sanguinis* |  | 5 |
| P19 | 19 | F | Skin soft tissue | *Parvimonas micra+Mogibacterium pumilum+Filifactor alocis+Prevotella oris+streptococcus intermedius* |  | 5 |
| P2 | 37 | M | Skin soft tissue | *prevotella intermedia+Parvimonas micra+Acinetobacter baumannii+streptococcus oralis+Fusobacterium nucleatum* |  | 5 |
| P5 | 69 | M | Abdominal | *Pseudomonas aeruginosa+Bacteroides thetaiotaomicron+prevotella intermedia+Clostridium leptum +Bacteroides distasonis* |  | 5 |
| P6 | 56 | M | Thorax | *Streptococcus constellatus+prevotella intermedia+porphyromonas endodontalis+Fusobacterium nucleatum+Catonella morbi* |  | 5 |
| P9 | 82 | F | Abdominal | *Enterococcus Faecium+Parabacteroides goldsteinii+Parabacteroides distasonis+Acinetobacter* |  | 4 |
| P12 | 74 | M | Abdominal | *Enterococcus faecium+Enterococcus faecalis+Bacteroides fragilis+Parabacteroides distasonis* |  | 4 |
| P26 | 79 | F | Thorax | *Olsenella uli+prevotella intermedia+Parvimonas micra+Viridans Streptococci* |  | 4 |
| P23 | 43 | M | Abdominal | *Stenotrophomonas maltophilia+Enterococcus avium+Enterococcus avium* |  | 4 |
| P1 | 49 | F | Abdominal | *Rahnella aquatilis+Alistipes+Phocaeicola vulgatus+Bacteroides fragilis* |  | 4 |
| P8 | 71 | M | Abdominal | *Fusobacterium nucleatum+Bacteroides heparinolyticus+Lactobacillus delbrueckii* | *Candida glabrata* | 4 |
| P11 | 43 | M | Abdominal | *Bacteroides fragilis+Bacteroides thetaiotaomicron+Parvimonas micra* |  | 3 |
| P13 | 44 | F | Thorax | *Fusobacterium nucleatum+Campylobacter rectus+Streptococcus constellatus* |  | 3 |
| P28 | 65 | F | Thorax | *Streptococcus anginosus+Streptococcus milleri+Klebsiella pneumoniae* |  | 3 |
| P29 | 61 | M | Thorax | *Neisseria bacilliformis+Prevotella oris* | *Candida albicans* | 3 |
| P30 | 60 | M | Skin soft tissue | *Streptococcus anginosus+Prevotella baroniae+Peptostreptococcus stomatis* |  | 3 |
| P31 | 43 | M | Skin soft tissue | *Aeromonas dhakensis+Enterococcus faecalis+ helcococcus kunzii* |  | 3 |
| P32 | 47 | M | Thorax | *Pseudomonas aeruginosa+Staphylococcus epidermidis+Nocardia cyriacigeorgica* |  | 3 |
| P20 | 35 | F | Abdominal | *Enterococcus Faecium* | *Candida albicans* | 2 |
| P21 | 55 | M | Abdominal | *Klebsiella aerogenes* | *Candida albicans* | 2 |

| **Supplementary Table 2**. The antibiotic adjustment after mNGS. | | | | | | | | |
| --- | --- | --- | --- | --- | --- | --- | --- | --- |
| **Patient ID** | **Age（years）** | **Sex** | **Site of infection** | **Bacteria** | **Fungus** | **The antibiotic before mNGS** | **The antibiotic after mNGS** | **The type of antibiotic adjustment** |
| P29 | 61 | M | Thorax | *Neisseria bacilliformis+Prevotella oris* | *Candida albicans* | Meropenem | Meropenem+Fluconazol | Initiating targeted therapy |
| P30 | 60 | M | Skin soft tissue | *Streptococcus anginosus+Prevotella baroniae+Peptostreptococcus stomatis* |  | Imipenem+Linazolamide | Cefoperazone sulbactam+Clindamycin | De-escalating therapy. |
| P33 | 68 | M | Thorax | Enterococcus avium |  | Cefoperazone sulbactam | Cefoperazone sulbactam+Fluconazol | Initiating targeted therapy |
| P24 | 52 | M | Skin soft tissue | Burkholderia melioides |  | Cefoperazone sulbactam+Imipenem+vancomycin | vancomycin+Voriconazole+Ceftazidime | Initiating targeted therapy |
| P14 | 62 | M | Enterocoelia | *Bacteroides thetaiotaomicron+bacteroides fragilis+Alistipes+Fusobacterium varium+Bilophila wadsworthia+Klebsiella pneumoniae* |  | Imipenem+Teicoplanin | Cefminox sodium | De-escalating therapy. |
| P23 | 43 | M | Abdominal | *Stenotrophomonas maltophilia+Enterococcus avium+Enterococcus avium* |  | Imipenem | Imipenem+Fluconazol | Initiating targeted therapy |
| P1 | 49 | F | Abdominal | *Rahnella aquatilis+Alistipes+Phocaeicola vulgatus+Bacteroides fragilis* |  | vancomycin | Tigecycline | Initiating targeted therapy |
| P5 | 69 | M | Abdominal | *Pseudomonas aeruginosa+Bacteroides thetaiotaomicron+Prevotella intermedia+Clostridium leptum +Bacteroides distasonis* |  | Teicoplanin | Cefminox sodium | De-escalating therapy. |
| P8 | 71 | M | Abdominal | *Fusobacterium nucleatum+Bacteroides heparinolyticus+Lactobacillus delbrueckii* | *Candida glabrata* | Imipenem | Minocycline+Fluconazol | Initiating targeted therapy |
| P9 | 82 | F | Abdominal | *Enterococcus Faecium+Parabacteroides goldsteinii+Parabacteroides distasonis+Acinetobacter* |  | Metronidazole+Cefoperazone sulbactam | Imipenem+Linazolamide | Initiating targeted therapy |
| P26 | 79 | F | Thorax | *Olsenella uli+Prevotella intermedia+Parvimonas micra+Viridans Streptococci* |  | Vancomycin | Cefoperazone sulbactam | Initiating targeted therapy |
| P31 | 43 | M | Skin soft tissue | *Aeromonas dhakensis+Enterococcus faecalis+ Helcococcus kunzii* |  | Clindamycin | Cefoperazone sulbactam+Linazolamide | Initiating targeted therapy |
